# Letter from Professor Martins

**Published:** 1840-05-16

**Authors:** 


To the Editors of the Medical Examiner.

### FOREIGN CORRESPONDENCE.

### LETTER FROM PROFESSOR MARTINS.

### No. VI.

*Course of General Pathology by Professor Andral—Lecture on Cancer.*

Paris, 25th March, 1840.

To the Editors of the Medical Examiner.

Tubercle is a product, without analogy in the healthful condition, which is developed under the influence both of general and local causes,—and the same may be said of cancer. For a long time it was very ill defined, and its name is merely an allusion to the *devouring* character of its progress. Tubercle is merely a product to which the name of tissue cannot be applied. Cancer, on the contrary, is composed of two abnormal tissues, united or isolated, to which the name of cancer is indifferently applied. These two tissues are, first, the *scirrhous;* secondly, the *encephaloid* or *cerebriform* tissues. After tubercle, cancer is the disease which carries off most victims; it is developed in every tissue and every organ. However, the lungs, the favourite seat of tubercle, are very rarely primitively affected with cancer, while it is often found in the liver, where tubercle so rarely shows itself.

The characters of the scirrhous tissue are the following: it is hard, grating under the scalpel, of a bluish white, semi-transparent, or transparent; its mass is not homogeneous, but divided into lobules separated by fibrinous intersections, and of a duller colour. Finally, it is without vessels. As the disease advances, (which is unfortunately the usual occurrence,) it loses its consistency, softens, becomes gelatiniform, or glue-like, and destroys itself by loss of substance, and ulceration. In the midst of these ravages, its fibrinous walls remain intact. Under the name of scirrhus has not always been comprised the tissue of which we speak. In the celebrated memoirs of the Academy of Surgery, organs indurated after chronic inflammation are designated under the name of scirrhus; we often see spoken of scirrhous lungs, which can be explained only by giving to the word scirrhus a different signification from that which we attach to it.

The encephaloid, or cerebriform tissue, has been so named by Laennec; but this appellation is correct only in certain stages of its development, when its consistence resembles that of the brain. Surgeons sometimes call it *medullary fungus.* Its characters, and mode of development, are the following: it is at first hard, without, however, creaking under the scalpel, of a milk-white colour, opaque, containing a great number of vessels. The latter are here developed as in false membranes, and in the normal membrane which surrounds the yolk of the egg. This development may be followed step by step. When a tissue of the kind is injected, it is soon perceived that it contains many more arteries than veins. This is owing, as has been shown by Professor Berard, to the fact that many veins are filled with encephaloid matter, which prevents the entrance of the injected liquid. This encephaloid matter is found not only in the veins of the tissue itself, but also in the surrounding veins which are connected with it. These anatomical peculiarities cannot be verified throughout; however, M. Andral has seen the veins of the liver strongly distended with the encephaloid tissue. After the encephaloid tissue has existed for a length of time, there is detected in it a liquid matter, of a milk-white colour, often not very apparent, and only to be squeezed out by pressure. This is what should be called encephaloid matter; it is this which may be absorbed, and give rise to secondary cancer. When this liquid is not very abundant, cancer is still in the state of crudity; but at the end of months, or perhaps years, it softens, owing to the more abundant secretion of the liquid matter, becomes congested, inflames like a healthy tissue, its numerous vessels are ruptured; hæmorrhage, more or less frequent, supervenes, which may be slight, or very abundant, and sometimes mortal. In cancer of the breast, in some cases, these hæmorrhages are seen to supervene before the appearance of any ulceration; the blood oozes out by little holes, almost imperceptible; then the encephaloid mass is reddish, fungous, and many authors term it *fungus hæmatodes*—a name also applied to erectile tumours. At this stage, ulceration takes place; this ulceration is never cured, but extends its ravages, the tissues which it reaches being converted into the encephaloid tissue. A few instances, however, unfortunately very rare, have been seen, in which a modification of the laws of nutrition brings about cicatrization. At other times the encephaloid mass passes into the state of gangrene, and detaches itself: this termination is not more common than the other. Instead of forming a separate mass, the cancerous matter may infiltrate the tissues; and this species of cancer is termed *cancer by infiltration.*

In the midst of the two tissues which constitute cancer, namely scirrhus and the encephaloid tissue, other substances may appear; but they are merely accidental, and no constituent parts of cancer itself. Such are melanosis (melanotic cancer) of the cartilages, and of the osseous and cartilaginous masses.

The solids, in the midst of which cancerous matter is deposited, may be affected by it in various manners. They may be pushed aside, destroyed, or transformed into cancerous substance. Cancer may be developed everywhere in the sub-cutaneous, sub-mucous, intermuscular, parenchymatous cellular tissue. It is rarely primitive in the coats of the blood-vessels; it often even spares the large arteries and veins, merely compressing them. It is thus that we see the aorta surrounded by a cancerous mass, without itself becoming so. The large veins are oftener affected than the arteries. Cerebriform matter, as we have seen, is found in the interior of the veins. Some, influenced by theoretic views, see, in this, blood transformed into cancerous matter; others, cancerous matter absorbed. This latter opinion, without being definitively proved, is the more probable. The lymphatic system is often affected. However, primitive cancer of the lymphatic glands is rare; but it often shows itself in them consecutively. The following is a remarkable instance of the ravages of cancer upon the lymphatic system. A lady had a very small cancer of the tongue. It was removed: there was no relapse. But, after the lapse of some time, the glands of the neck became cancerous, and formed an enormous mass, which carried off the patient. Cancer of the nervous tissue presents itself usually under the encephaloid form in the nerves, and especially in the brain. In the muscular tissue, it is consecutive, never primitive; in fact, this tissue undergoes no transformation, but its fibres become pale and atrophied without changing in character. Cancer of the tongue is developed in the sub-mucous cellular tissue. This is the tissue of all others in the economy most liable to its attacks. It may also be developed in the mucous tissue itself, under the form of vascular, encephaloid tubercles, or vegetations, resembling in vascularity the tissue in which they originate. A form of cancer shows itself in the skin, which is peculiar to it: it consists of little flat excrescences, which remain long indolent, and end by softening and ulcerating, often with very rapid progress, (Lupus Esthiomène.) The serous tissue is rarely primitively affected: the cancerous masses of the omenta originate in the sub-serous cellular tissue. In the bones, cancer may have three different points of origin: first, in the body itself of the bone; secondly, in the medullary membrane; thirdly, between the external periosteum and the bone. Cancer of the thyroid gland is rare, and I know of no authentic example of its having originated in the pulmonary tissue; this latter it attacks only consecutively, and shows itself under the form of round, encephaloid masses, scattered through the parenchyma of the organ. It is often primitive in the liver; when it appears here consecutively, it is consequent on scirrhus of the stomach. The spleen, kidneys, ovaries, neck of the uterus, penis, may all be affected with cancer. The disease in the ovaries is rare, in the uterus very common, but nowhere so much so as in the mammæ.

Cancer often developes itself at several points simultaneously, and travels, so to say, through the economy: this is specially true with regard to the encephaloid tissue. In this point of view, we distinguish three several varieties of progress: first, several parts may be simultaneously attacked, without the possibility of pointing out any well-marked point of departure; secondly, an isolated part may be attacked, and the neighbouring ganglia become consecutively infected; thirdly, after a part is attacked, the neighbouring ganglia become involved, and then all the organs. Very often immediately after the surgeon has removed a cancer, the disease is seen to develope itself at the same time throughout the system, then to reproduce itself in the very point where the operation was performed.

There certainly exists a general cause which occasions the production of cancer, but it is difficult to define it. To a certain point, we are able to diagnosticate in certain persons a diathesis favourable to the development of tubercle; but this is not the case with cancer. It appears alike in all constitutions. In opposition to tubercle, it frequently developes itself after the age of thirty-five, and is quite rare after sixty-five. There are two exceptions to this rule; namely, cancer of the eye, and the cancer of chimney-sweeps.*

Inflammation cannot contribute to the de-

*I may add a third, the variety of cancer of the face, known under the term *lupus*, which is often noticed before the age indicated by M. Andral.

velopment of cancer, but it may determine its appearance in one point rather than another. Many women receive blows on the breast, are affected with mammary inflammation and abscesses, without their becoming cancerous. In others, this horrible disease declares itself without any known developing cause. Its progress is usually chronic; it has been seen to spread with a rapidity equal to that of acute diseases, and in these cases its termination is almost always fatal.

